# Characterization of the complete chloroplast genome of *Tulipa iliensis* (Liliaceae)

**DOI:** 10.1080/23802359.2020.1773333

**Published:** 2020-06-08

**Authors:** Xiuting Ju, Guomin Shi, Zhiqiang Hou, Chaohai Wu, Gaofeng Liu, Caixia Cao, Nan Tang

**Affiliations:** a College of Agriculture and Animal Husbandry, Qinghai University, Xining, China; b The Key Laboratory of Landscape Plants of Qinghai Province, Xining, China; c State Key Laboratory of Plateau Ecology and Agriculture, Qinghai University, Xining, China

**Keywords:** *Tulipa iliensis*, Liliaceae, chloroplast genome

## Abstract

The chloroplast genome and evolutionary relationship analysis of *Tulipa gesneriana* L. could provide fundamental genetic reference for its molecular breeding and biological research. The complete chloroplast genome of *Tulipa iliensis* was sequenced and reported here. Its chloroplast genome was 151,744 bp in length, containing a pair of inverted repeated regions (26,354 bp) which were separated by a large single copy region of 81,794 bp, and a small single copy region of 17,242 bp. Moreover, a total of 133 functional genes were annotated, including 87 mRNA, 38 tRNA genes, and 8 rRNA genes.The phylogenetic relationships of 16 species indicated that *T. iliensis* was closely related to *T. altaica*.

Tulip, belonging to the genus Tulipa L. in the family Liliaceae. Most varieties of garden tulips are belonging to *Tulipa gesneriana* (Okazaki and Nishimura 2000). Some wild Tulipa species native to China, which have yellow, vivid flowers and strong adaptability (Han et al. 2014). Wild Tulipa species constitute a potential genetic resource for the improvement in tulip cultivars through interspecific hybridization because they have some favorable genes, such as tolerance to drought and cold, resistance to diseases and viruses (Xing et al. 2020). Here, we sequenced the complete chloroplast genome of *Tulipa iliensis* could provide fundamental genetic reference for its molecular breeding and biological research.

In this study, *T. iliensis* were collected from Altay Region, Buerjing County, Xinjiang Province, China (48°41′48″N, 87°02′03″E) in September 2018. The specimen was kept in the Key Laboratory of Landscape Plants of Qinghai Province, Qinghai University, Xining, China (accession number: JXT-2018-YJX017). Genomic DNA was extracted using leaves from the same plant. Genomic sequencing was performed on the Illumina HiSeq Platform (Illumina, San Diego, CA) with a read length of 150 bp. The software SPAdes v.3.14.0 (Bankevich et al. 2012) was employed to assemble the chloroplast genome. Then, Prodigal v2.6.3 (https://www.github.com/hyattpd/Prodigal), Hmmer v3.1b2 (http://www.hmmer.org/) and Aragorn v1.2.38 (http://130.235.244.92/ARAGORN/) were respectively used to annotate the coding sequences (CDs), transfer RNA (tRNA) genes and ribosomal RNA (rRNA) genes.

The complete chloroplast genome of *Tulipa iliensis* was 151,744 bp in length with a typical quadripartite structure, containing a pair of inverted repeated (IR) regions (26,354 bp) that are separated by a large single copy (LSC) region of 81,794 bp, and a small single copy (SSC) region of 17,242 bp. The GC content of the whole complete chloroplast genome was 36.64%. A total of 133 functional genes were annotated, including 87 proteincoding genes (mRNA), 38 tRNA genes, and 8 rRNA genes. The protein-coding genes, tRNA genes, and rRNA genes account for 65.41, 28.57, and 6.02% of all annotated genes, respectively.

Based on chloroplast genomes assembled here and downloaded from GenBank, phylogenetic relationships of 14 Liliaceae species were resolved by means of Neighbor-joining with 2 species from Amaryllidaceae as outgroup (Figure 1). After aligned using MAFFT (Katoh and Standley 2013), the Neighbor-joining tree was built using MEGA7 (Kumar et al. 2016) with bootstrap set to 1000. In the phylogenetic tree, *T. buhseana* and *T. altaica* went to an independent clade with a 100% node support rate.

**Figure 1.**
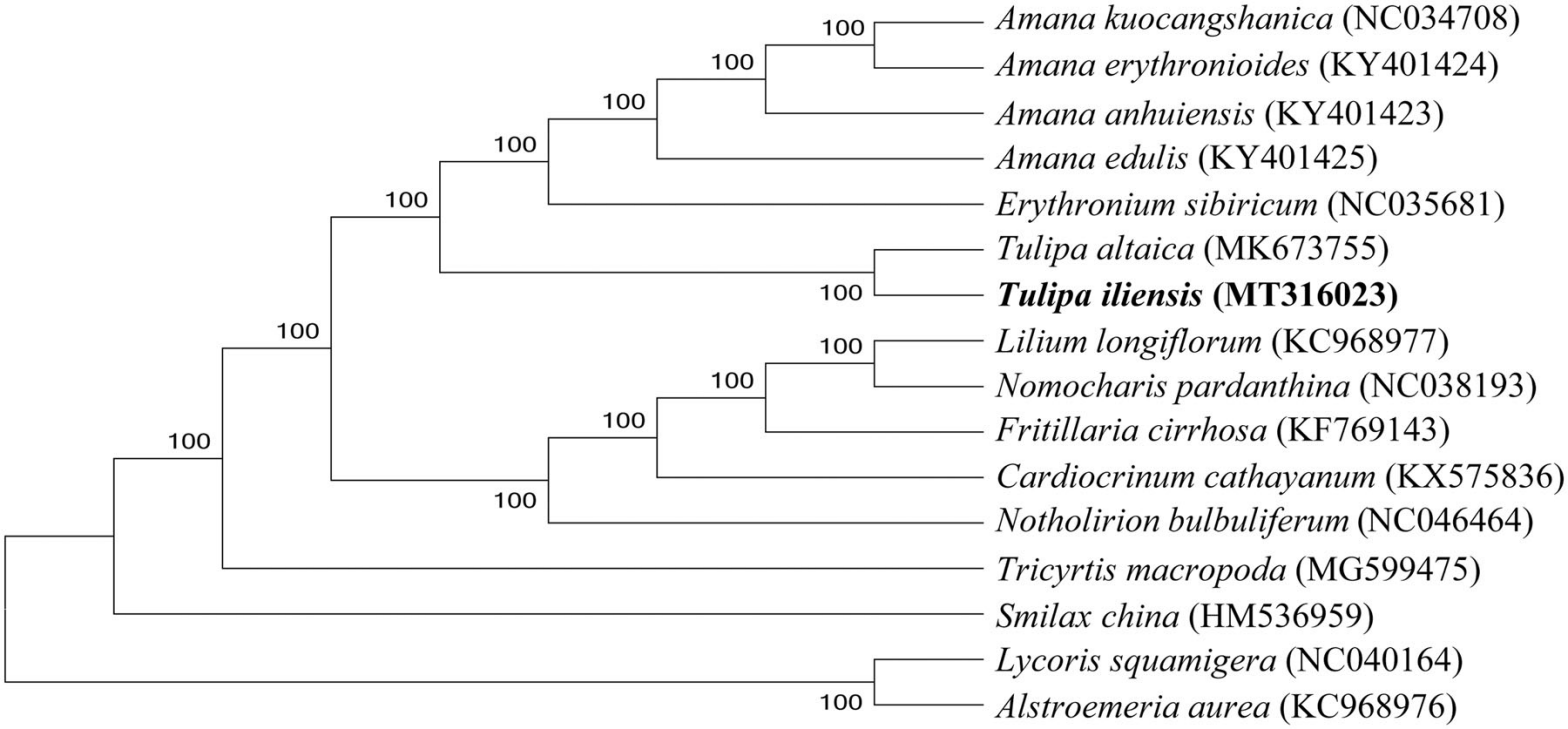
Phylogenetic relationships of 16 species based on complete chloroplast genome using the neighbor-joining methods.

## Data Availability

The data that support the findings of this study are openly available in Genbank at https://www.ncbi.nlm.nih.gov/genbank/, reference number MT316023.

## References

[CIT0001] Bankevich A, Nurk S, Antipov D, Gurevich AA, Dvorkin M, Kulikov AS, Lesin VM, Nikolenko SI, Pham S, Prjibelski AD, et al. 2012. SPAdes: a new genome assembly algorithm and its applications to single-cell sequencing. J Comput Biol. 19(5):455–477. 22506599 10.1089/cmb.2012.0021 PMC3342519

[CIT0002] Han BX, Zhang K, Huang LQ. 2014. Amana wanzhensis (Liliaceae), a new species from Anhui China. Phytotaxa. 177(2):118–124.

[CIT0003] Katoh K, Standley DM. 2013. MAFFT multiple sequence alignment software version 7: improvements in performance and usability. Mol Biol Evol. 30(4):772–780. 23329690 10.1093/molbev/mst010 PMC3603318

[CIT0004] Kumar S, Stecher G, Tamura K. 2016. MEGA7: Molecular Evolutionary Genetics Analysis version 7.0 for bigger datasets. Mol Biol Evol. 33(7):1870–1874. 27004904 10.1093/molbev/msw054 PMC8210823

[CIT0005] Okazaki K, Nishimura M. 2000. Ploidy of progenies crossed between diploids, triploids and tetraploids in tulip. Acta Hortic. 522(522):127–134.

[CIT0006] Xing GM, Qu LW, Zhang W, Zhang YQ, Yuan XF, Lei JJ. 2020. Study on interspecific hybridization between tulip cultivars and wild species native to China. Euphytica. 216(4):66.

